# Body awareness in Goffin’s cockatoos (*Cacatua goffiniana*)

**DOI:** 10.1038/s41598-026-57500-7

**Published:** 2026-09-17

**Authors:** Paula Hundegger, Remco Folkertsma, Rachel Dale, Alice M. I. Auersperg, Antonio J. Osuna-Mascaró

**Affiliations:** 1 Comparative Cognition, Messerli Research Institute, University of Veterinary Medicine Vienna, Medical University Vienna, University of Vienna, Veterinaerplatz 1, 1210 Vienna, Austria; 2 Department for Psychosomatic Medicine and Psychotherapy, University for Continuing Education Krems, Krems an der Donau, Austria

**Keywords:** Ecology, Ecology, Zoology

## Abstract

Mirror self-recognition (MSR) is widely used to assess self-awareness, but its reliance on vision may limit its suitability for cross-species comparisons. A complementary approach is the body-as-obstacle (BAO) paradigm, which tests whether individuals recognize their own body as a causal impediment to solving a task. Although BAO has been applied in mammals, it has not previously been adapted for birds. Here, we tested 15 Goffin’s cockatoos (*Cacatua goffiniana*) in an avian BAO task in which subjects had to retrieve and pass an object to an experimenter. In the test condition, the object was attached to the mat on which the bird stood, requiring subjects to step off the mat to succeed; two control conditions were unattached and attached-to-ground. A pre-test controlled for stepping off due to mat movement or discomfort. Cockatoos passed the pre-test (mean success ± SE: 99.4 ± 0.6%) and stepped off markedly more often in the test condition (97.8 ± 0.9%) than in the unattached (0.7 ± 0.3%) or attached-to-ground control (39.1 ± 3.9%). These findings provide the first application of the BAO paradigm in birds and support the use of comparative, multimethod approaches to body awareness.

**Supplementary Information:**

The online version contains supplementary material available at https://doi.org/10.1038/s41598-026-57500-7.

## Introduction

Since Gallup’s pioneering study of chimpanzees^1^ and Amsterdam’s later adaptation for children^2^, the mirror self-recognition (MSR) test has been widely regarded as the primary method for assessing self-awareness across species. Although the MSR task has been highly influential, its comparative value has been widely disputed because it relies heavily on vision and may therefore be less ecologically valid for species with different dominant sensory modalities. Moreover, reports of impressive MSR-like behavior in a relatively small-brained animal such as the cleaner wrasse (*Labroides dimidiatus*)^3^ have sparked debate over what the test actually captures. This prompted a shift towards a gradualist, modular framework that accounts for species-specific sensory modalities and ecological contexts^4–6^. As de Waal argued, animals must represent the affordances of their own bodies in relation to the physical and social environment, suggesting that mirror responses alone should not remain the sole benchmark for studying self-awareness^6^. A highly promising complementary approach is the “body-as-an-obstacle” (BAO) task. Unlike MSR, which focuses on visual self-recognition, BAO assesses whether subjects treat their own body as a causal obstacle during goal-directed action.

The BAO task measures an individual’s ability to recognize parts of their own body as an impediment to achieving a goal, thereby assessing body-related self-representation. Developed for human infants^7,8^, the task requires subjects to hand over an object while standing on a mat directly attached to the object; success requires stepping off the mat to eliminate their own body weight as the source of obstruction. In a control condition, the obstruction is external (e.g., a heavy object), so stepping off is unnecessary. In humans, BAO performance emerges at roughly the same age as MSR (18–24 months) and correlates moderately with it^7,9^. Cross-cultural work shows that infants from autonomy-oriented cultures tend to pass MSR earlier, whereas those from relatedness-oriented cultures succeed earlier on BAO^9^. These findings indicate that MSR and BAO follow distinct developmental trajectories and rely on distinct processes. At the same time, they represent different modes of self-representation. Because BAO targets the embodied self, it offers a valuable alternative for species in which vision is not the dominant sensory modality.

From a cognitive standpoint, both MSR and BAO require causal reasoning, but they differ in sensory demands. MSR depends on recognizing visual contingencies and features, whereas BAO relies on awareness of direct body-environment interaction without requiring an external visual representation^7,9^. Because BAO lacks visual prerequisites, it does not require prior mirror experience or visual inspection of the body, which may render it more ecologically valid for animals that rely primarily on non-visual modalities^10^. These advantages have motivated recent applications of BAO to non-human species.

Researchers have increasingly used the BAO paradigm to assess body awareness in nonhuman animals, with positive results in elephants, dogs, chimpanzees, and lowland gorillas^10–12^. Because elephants’ size and strength precluded an “impossible” control, Dale and Plotnik included an unattached control and a foot-discomfort control; elephants stepped off the mat significantly more often in the test than in either control, whereas the two controls did not differ significantly from one another^11^. In dogs, Lenkei et al. implemented both controls and an attached-to-ground condition, and dogs again left the mat more often and sooner in the test than in the critical attached control, including in the first trial^10^. Vanhooland et al. later adapted BAO for great apes using a non-social design and a single control analogous to the unattached condition used in earlier studies^12^.

Together, these three studies showed that success on BAO, although correlated with MSR in humans, can be passed independent of having passed MSR in these animals. Adult Asian elephants and great apes have demonstrated MSR under certain conditions (e.g., after appropriate prior experience;^13–15^), whereas dogs have not^16^, suggesting that body awareness may be evolutionarily widespread and may represent a fundamental, ecologically relevant component of self-representation.^4^

Although BAO has not yet been adapted for birds, there are strong reasons to do so. Birds, particularly parrots and corvids, have demonstrated a cognitive toolkit comparable to that of great apes^17,18^, yet they have often yielded inconsistent evidence under MSR paradigms (e.g., replication attempts have yielded different results^19,20^). This evidence, together with behavioral and neuroanatomical findings often linked to MSR in mammals^21–23^, has led several authors to advocate avian species as compelling non-primate candidates for testing self-awareness using better-adapted methodology^5^.

Goffin’s cockatoos (*Cacatua goffiniana*) are highly innovative problem-solvers: they flexibly manufacture, modify, and use tools, and they engage extensively in haptic exploration with their beak and feet^18,24–26^. This tactile interaction style aligns well with the embodied, action-based demands of BAO. Like other parrots tested thus far, Goffin’s cockatoos have failed in attempts to test MSR^27^.

However, sulfur-crested cockatoos have been observed opening rubbish bins in a manner reminiscent of BAO-type problem-solving^28^, although the learning mechanism remains unknown: they have learned to step off the lid of a rubbish bin onto the rim of the bin, avoiding the impediment of their own body weight for opening the lid.

These observations suggest that other, highly flexible white Cacatua species, such as the Goffin’s cockatoos, are strong candidates for BAO testing.

**Fig. 1:**
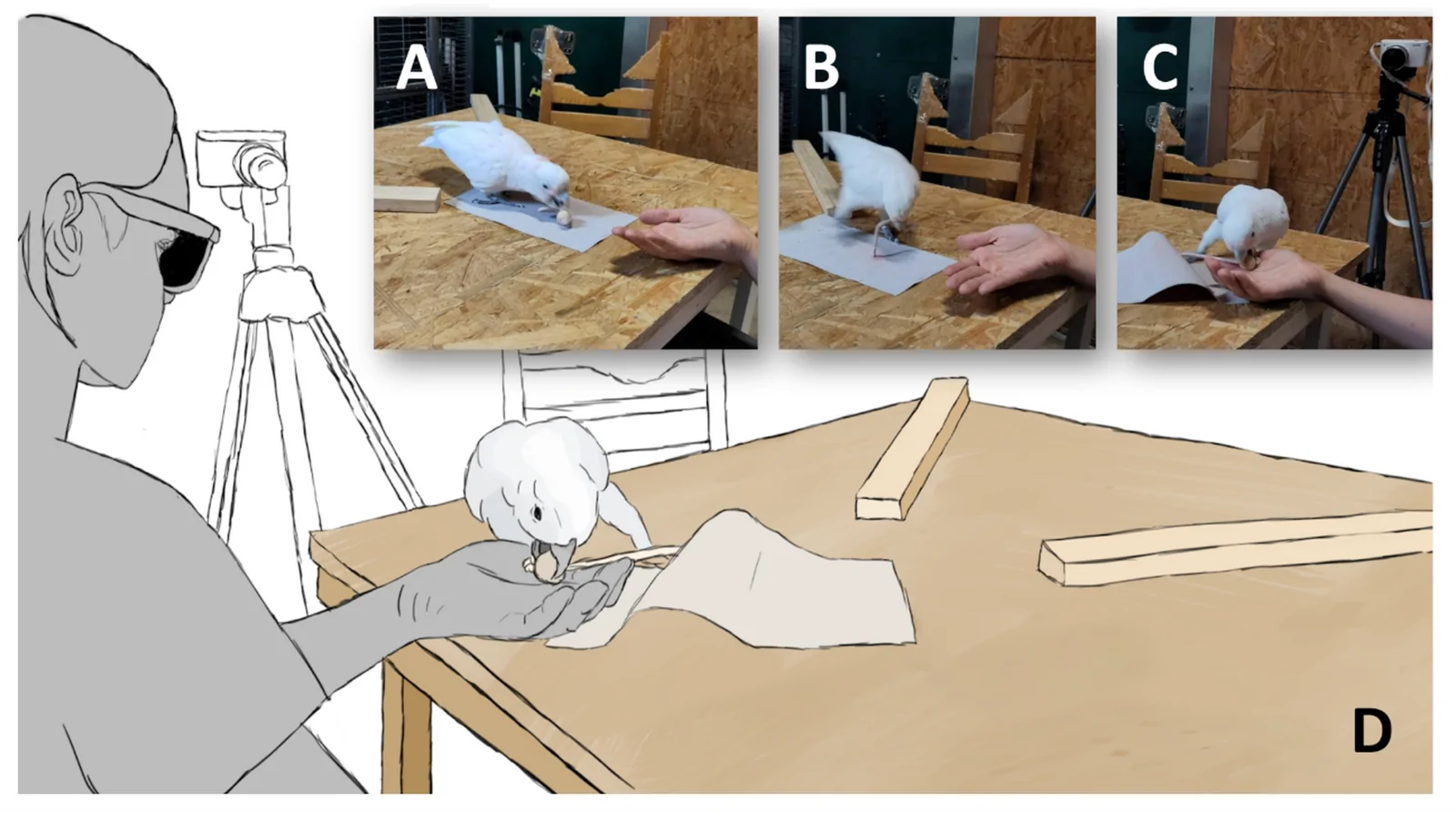
Setup and testing procedure during the test condition: (**A**) picking up the ball after the “give-me” command, (**B**) pulling and stepping out of the mat, (**C**) transferring the ball while out of the mat. (**D**) schematic representation of the setup during the transfer sequence (including mat, corridor and left camera).

Here, we present the first avian adaptation of the BAO paradigm, using Goffin’s cockatoos as a model species (Fig. 1; Supplementary Video S1). We employ a “give-me” command to preserve the social element of the original design and include the key control comparisons used in mammalian BAO studies (unattached and attached-to-ground), plus a pre-test controlling for foot discomfort. We conceal visual differences between test and control conditions to prevent cue-based anticipation, and we focus the analysis on the first test session to reduce the likelihood that the main condition effect reflects repeated exposure across sessions. We hypothesize that Goffin’s cockatoos would be able to treat their own body as a causal obstacle to task completion in a body-as-obstacle task.

## Results

### Pre-test performance

All fifteen Goffin’s cockatoos successfully passed the pre-test. Out of 180 pre-test trials (15 subjects × 12 trials), 179 were completed successfully, corresponding to a success rate of 99.4%. Only one bird stepped off the mat during a pulling attempt; however, because this individual completed all remaining pre-test trials successfully, all subjects were retained for the main experiment.

### Overall performance across conditions

Across all experimental sessions, cockatoos stepped off the mat in the test condition in 97.8 ± 0.9% of trials (mean ± SE across subjects; 528/540 trials overall). In contrast, they stepped off in only 0.7 ± 0.3% of trials in the unattached control (4/540 overall) and 39.1 ± 3.9% of trials in the attached-to-ground control (211/540 overall).

**Fig. 2:**
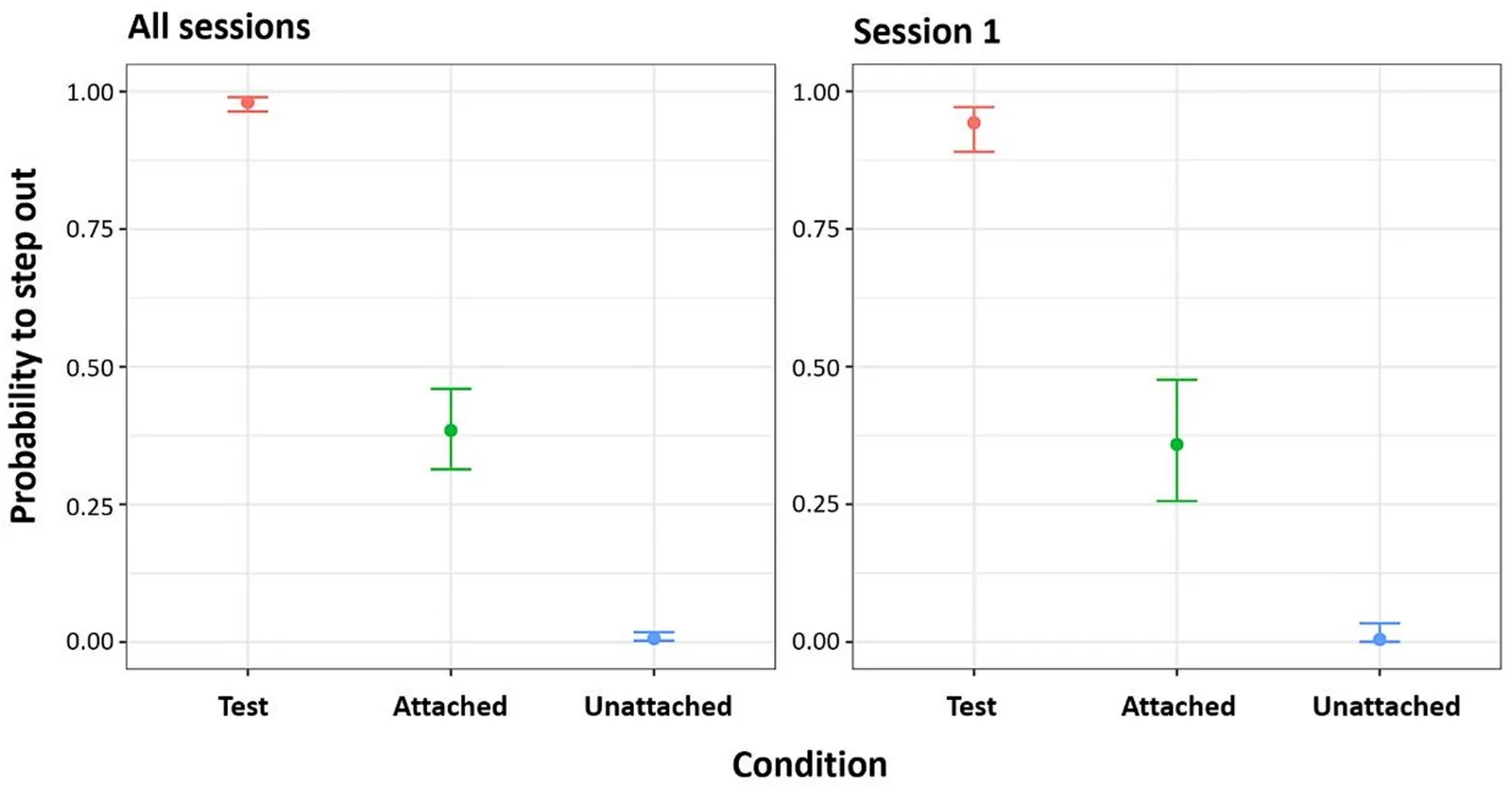
Probability of stepping off the mat across test and control conditions: Model-estimated probability of stepping off the mat (95% confidence intervals) in Goffin’s cockatoos under three conditions: Test (object attached to the mat on which the bird stood), Attached (attached-to-ground), and Unattached (object not attached). The left panel shows estimates for all sessions, and the right panel shows estimates for Session 1 only.

Birds were substantially more likely to step off the mat in the test condition than in either control condition (Fig. 2). A binomial GLMM showed a significant effect of condition on step-off probability (likelihood-ratio test: χ^2^ (2) = 88.85, *p* < 0.001). Post hoc pairwise comparisons confirmed that the test condition differed significantly from both the attached-to-ground and unattached controls, and that the two control conditions also differed (all adjusted *p* < 0.001). The grouping factor (subject group identity) did not significantly affect step-off probability (*p* = 0.92), suggesting that group assignment (see methods section) was unlikely to account for the observed pattern.

### Behavioral responses in the attached-to-ground control condition

In the attached-to-ground control condition, birds could step off the mat while still holding the ball, but stepping off could not solve the task. Because this condition was unsolvable by design, trials were not classified according to successful object transfer. Instead, we recorded whether the bird disengaged from the unsuccessful attempt within the trial window. Behaviors counted as disengagement included walking away, redirecting activity, or showing signs of frustration. Across all sessions, 329 of 540 attached-to-ground trials (60.9%) met this disengagement criterion. Among trials that ended in disengagement, the most common outcome was walking away (158/329, 48.0%), whereas the remaining disengagement trials mainly involved redirected contact behaviors during unsuccessful attempts to obtain the object. Contact with the foot ring was rare (18/329, 5.5%). Failures in this condition most often involved the subject stepping off the mat and continuing to pull, mimicking their actions in the test condition. Only a small number of trials failed due to time expiration (16 out of a total of 540).

### First-session analysis

During the first session, cockatoos stepped off the mat in the test condition in 93.3 ± 2.7% of trials (168/180 overall), compared with 0.6 ± 0.6% in the unattached control (1/180 overall) and 37.2 ± 4.8% in the attached-to-ground control (67/180 overall). A separate binomial GLMM showed a significant effect of condition on step-off probability (likelihood-ratio test: *χ*^*2*^(2) = 29.40, *p* < 0.001). Post hoc pairwise comparisons confirmed that the test condition differed significantly from both control conditions, and that the two control conditions also differed (all adjusted *p* < 0.001). As in the overall analysis, the grouping factor did not significantly affect step-off probability. These results indicate that the condition-dependent pattern was already evident in the first session.

### Session consistency

A comparison of models with and without the fixed effect of session revealed no significant difference (likelihood ratio test: *χ*^*2*^(1) = 0.70, *p* = 0.403), indicating that performance remained stable across sessions. Thus, we found no evidence that the condition effect changed across sessions.

### Trial duration

Trial duration differed significantly between successful and unsuccessful test trials. Successful test trials were on average 19.2 s shorter than unsuccessful ones (*p* < 0.0001). Comparisons between successful trials across conditions also revealed significant effects: successful test trials were on average 2.8 s shorter than attached-to-ground trials that ended in disengagement (*p* < 0.001) and 2.6 s longer than successful unattached control trials (*p* < 0.0001), indicating that performance efficiency varied systematically across conditions.

## Discussion

In this study, we tested whether Goffin’s cockatoos represent their own body as a causal obstacle in a body-as-obstacle task. Consistent with our hypothesis, cockatoos stepped off the mat significantly more often in the test condition, where their own body impeded task success, than in either control condition. This robust pattern across individuals and sessions provides strong evidence that the birds distinguished between situations in which their body was causally involved in the problem and those in which it was not.

Some alternative explanations for this behavior can be ruled out. Firstly, the near-perfect performance in the pre-test suggests that stepping off the mat was not a default response to mat movement or discomfort under the feet. The cockatoos had been previously habituated to standing on the mat while the experimenter pulled it, and the fact that almost no pre-test trial failed indicates that discomfort alone cannot account for the high step-off rates observed in the test condition. Secondly, repeated exposure across sessions is unlikely to explain the results. Performance in the first session closely matched that observed across all three sessions, and statistical comparisons revealed no significant effect of session. This pattern indicates that the condition effect was already present from the first session and remained stable across sessions, making it unlikely that the main result was driven by repeated exposure across sessions alone. However, because the present analyses did not model acquisition within session, we refrain from interpreting the performance as spontaneous innovation in the strict sense.

Task difficulty alone is unlikely to account for the observed pattern. While the attached-to-ground control elicited substantially more step-offs than the unattached control, the test condition still produced significantly higher step-off rates than either control. Because the attached-to-ground condition was unsolvable, some of this difference likely reflects persistence, frustration, or difficulty-related disengagement. Many birds nevertheless persisted in a manner consistent with previous reports of high motivation and persistence in this species^5,24,29^. The fact that step-off rates were highest when the bird’s own body caused the obstruction is consistent with sensitivity to bodily causality rather than difficulty alone. The trial-duration results are consistent with this interpretation. In the test condition, successful trials were markedly shorter than unsuccessful ones, and successful test trials were also shorter than attached-to-ground trials that ended in disengagement. This pattern suggests that when the problem could be solved by moving the body, birds often did so relatively efficiently, whereas the unsolvable attached-to-ground condition elicited more prolonged interaction before disengagement.

Behavioral observations in the attached-to-ground control condition further support this interpretation. Trials that ended in disengagement were characterized by behaviors such as walking away or redirecting activity, whereas failures often involved continued pulling attempts despite the constraint not being body-related. Frustration-related behaviors and redirected contact during the attached-to-ground control were consistent with sensitivity to an unsolvable external constraint while remaining motivated to complete the task. Although frustration is not itself evidence of body awareness, its systematic occurrence in the attached-to-ground control, but not in the test condition, suggests that birds differentiated between an external constraint and a bodily one.

Several limitations should be considered when interpreting these findings. Firstly, the attached-to-ground control was not fully symmetrical to the unattached control, because it required subjects to disengage from an unsolvable action rather than simply complete a solvable one. This difference in task difficulty likely contributed to the intermediate rate of step-offs observed in that condition. Secondly, the interpretation of outcomes in the attached-to-ground control depended partly on classifying disengagement behaviors as giving up; although these behaviors were defined a priori, this criterion necessarily involves some degree of behavioral interpretation. Finally, body-as-obstacle paradigms and mirror self-recognition tasks do not measure identical aspects of self-representation, and success in the present task should therefore not be interpreted as equivalent to mirror self-recognition. The pre-test was designed to rule out stepping off in response to mat movement or discomfort alone, but it did not reproduce the exact force geometry of the test condition. We therefore cannot fully exclude the possibility that differences in pulling direction or force configuration contributed to behavior. However, because birds also differentiated strongly between the test and attached-to-ground conditions, force geometry alone is unlikely to account for the full pattern.

Our findings are particularly informative in light of previous mirror self-recognition (MSR) studies in Goffin’s cockatoos. Although these birds did not pass the classic MSR test, there were behaviors potentially indicative of contingency checking^27^. The present results demonstrate that the absence of mirror self-recognition does not necessarily preclude other forms of self-representation. Similar discrepancies have been reported in dogs and Asian elephants, which succeed in body-as-obstacle paradigms but differ in MSR performance^10,11^. Together, these findings reinforce the growing consensus that MSR captures only a narrow, visually mediated aspect of self-recognition and should not be treated as a universal benchmark.

The reliance on body-based and haptic information observed here aligns well with what is known about Goffin’s cockatoos’ problem-solving strategies. Previous work indicates that these birds frequently prioritize tactile and proprioceptive feedback over visual cues when interacting with objects^5,24^. Body awareness tasks, unlike mirror-based tasks, do not require learning a correspondence between an external visual image and the self. Instead, they draw on sensory information that is continuously available during everyday action. Recent work on mirror self-recognition has argued that when self-recognition emerges rapidly, too quickly to plausibly depend on associative learning, it is more parsimoniously interpreted as reflecting a pre-existing capacity rather than one constructed through experience^30^. In this context, the early emergence of the condition effect in our body-as-obstacle paradigm is consistent with the possibility of pre-existing self-representational abilities. Such abilities may align with observations of wild sulfur-crested cockatoos opening rubbish bins in Sydney^28^. The manner in which they accomplish this may involve related body-object coordination demands similar to the behavior observed in the test condition of our study.

More broadly, these results support the view that self-representation is not a unitary cognitive ability but rather a constellation of related processes that may vary across species and sensory modalities. Within an embodied cognition framework, self-representation emerges from the integration of bodily action, perception, and causal reasoning, shaped by species-specific ecological and evolutionary pressures. From this standpoint, the present findings highlight two key considerations for comparative cognition research. First, cognitive tests must be matched to the perceptual and motor affordances of the species under study. Second, employing a diverse set of paradigms is essential for capturing the multifaceted nature of self-representation.

In line with findings from dogs and Asian elephants, Goffin’s cockatoos successfully solved a body awareness task despite failing to meet the criteria of mirror self-recognition^10,11,27^. Asian elephants have demonstrated success in both paradigms, whereas other species show dissociations across tasks. These cross-species differences suggest that self-representation is not a single, monolithic capacity but rather a collection of related abilities that may rely on different sensory modalities and problem-solving strategies. Consequently, an exclusive focus on visually mediated paradigms such as MSR risks underestimating self-representational capacities in species for whom vision is not the dominant channel for interacting with the environment. From an evolutionary and embodied cognition perspective, investigating self-representation therefore requires methods that are tailored to a species’ ecological niche, morphology, and typical modes of interaction with the world. Taken together, our findings emphasize the importance of adopting a pluralistic methodological approach when studying self-recognition and highlight body-based paradigms as a powerful complementary tool for exploring self-representation across taxa.

## Methods

### Subjects

For this study, we tested 15 Goffin’s cockatoos from the Goffin Lab Goldegg in Lower Austria. The tested group consisted of eight males and seven females with ages ranging from 2 to 16 years. All birds were captive-born and purchased from accredited European breeders and could be individually identified using colored foot rings. They are permanently housed under environmentally and socially enriched conditions, with an indoor (45 m^2^; 3–6 m high) and outdoor (ca. 200 m^2^; 3–4.5m high) aviary. The birds at the Goffin lab have been the subjects of various previous studies, especially involving physical cognition but not in the context presented (BAO). All were already trained to pass an object on command. During the voluntary testing, each subject was individually called to the testing room, where the experiment took place and where no other birds could observe them.

### Ethics

All experiments were voluntary, appetitive, behavioral, and non-invasive, and as such, they do not fall under the classification of animal experiments as defined by the Austrian Animal Experiments Act. Nevertheless, the experiment was approved by the Ethics and Animal Welfare Committee of the University of Veterinary Medicine Vienna (ETK-175/11/2023). All methods were performed in accordance with the relevant institutional, national, and international guidelines and regulations. Written informed consent was obtained from the individuals whose faces are visible in Fig. 1 and Supplementary Video S1 for publication of identifiable images and video in an online open-access publication.

### Apparatus/general materials

For the main part of the experiment, we used a 15 × 26 cm grey mat made of a faux leather and linen material. For the test condition, a wooden ball with a diameter of 2 cm and a hole in the middle was attached to the mat with a linen cord. The same wooden ball, cord and mat materials were used for the control conditions. In the attached-to-ground control, the same ball-and-cord assembly was anchored to the table through a concealed attachment point; thus, the ball remained movable and could still be held in the beak, but pulling it could not solve the task because the obstruction was external rather than body-based. The whole experiment was done on a table, with a wooden platform specially designed to fit on top of it. Two wooden planks on the table formed a corridor which the subjects had to walk to start each trial from the same spot. A concealed hole allowed the object to be attached and secured when required while maintaining a visually similar setup across sessions. The pre-test took place on the same table with the same corridors and mat but instead of a wooden ball tied to a cord, we used a glass marble (diameter ca. 1 cm) and a small plastic bucket for the subjects to put the marble in (Fig. 1).

### Procedure

**Fig. 3:**
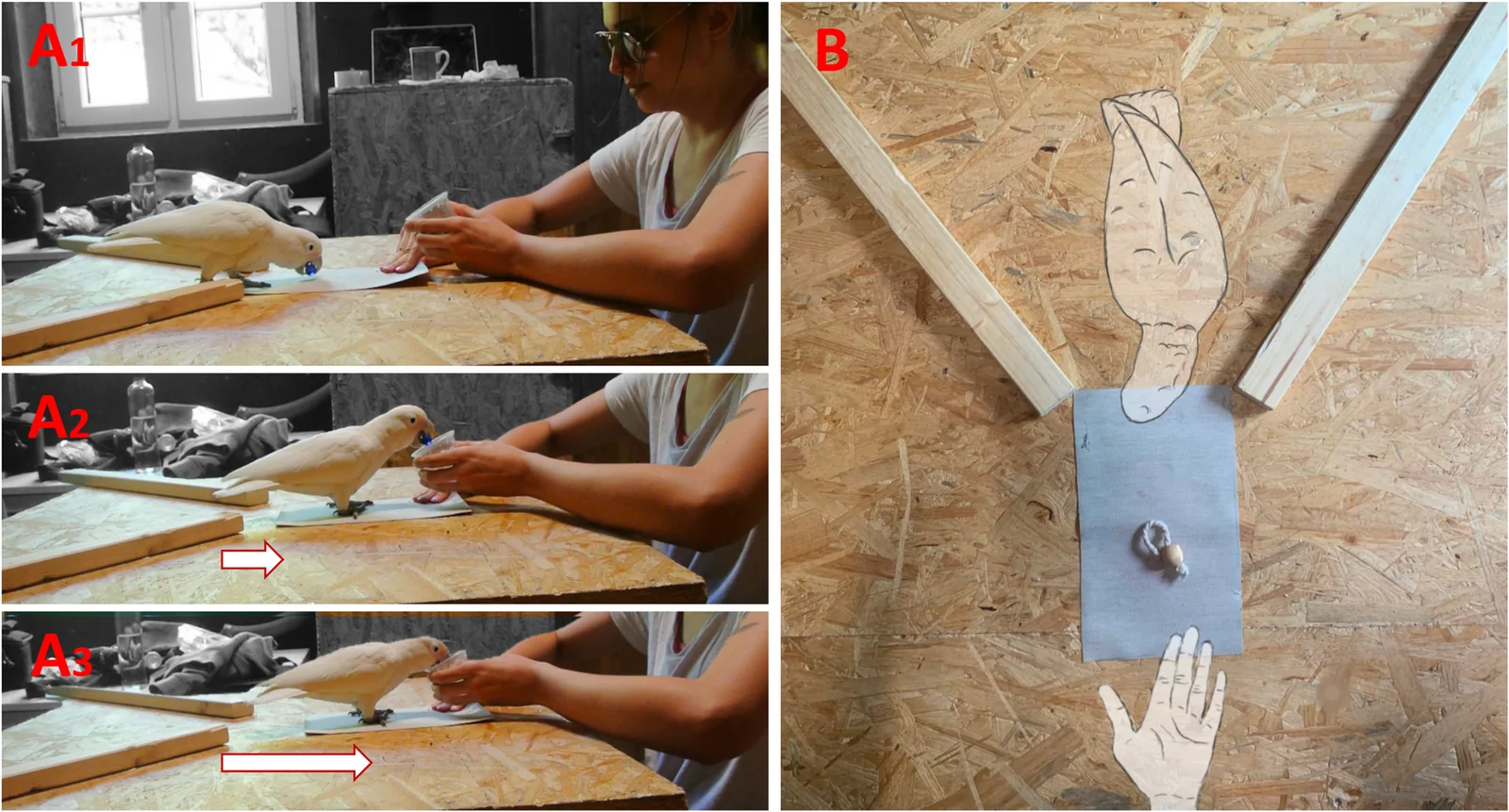
Pre-test procedure and general test setup: (**A1**) pre-test invitation to put the marble in the plastic cup, (**A2**) the researcher starts pulling immediately when the marble is lifted, (**A3**) the mat is pulled while the subject remains standing on it. (**B**) overview of the corridor, mat, ball and cockatoo, and researcher hand position.

All testing was conducted inside a visually occluded testing room in order to avoid social learning. *Training.* Fifteen subjects were habituated to the apparatus and learned to walk the corridor (Figs. 1, 3). Afterwards they repeated existing training in passing objects on command and, through repeated exposure, learned to not fear the moving of the mat for the pre-test.

*Pre-test.* The purpose of this pre-test was to reduce the likelihood that stepping off in the test condition was driven by mat movement or discomfort alone. While the subject was engaged in a separate task (placing a marble in a plastic cup), the researcher pulled the mat to expose the bird to mat movement underfoot without requiring object-directed pulling. (Fig. 3A).

*Test condition.* The experimental test condition started with the cockatoo on top of the mat. The bird was then told to pass an object, which was attached to the mat, to the experimenter. The trial ended either when the subject stepped off the mat to pick up the object and pass it to the experimenter (success), when the subject walked away (failure) or after 30 s of failed attempts (failure).

*Control conditions.* Additionally, there were two control conditions to exclude alternative explanations for stepping off the mat: In the first, the subject was told to pass an object that was not attached to anything. This was to test if the subject could distinguish whether or not it was necessary to get off the mat in order to pass the object. In the second control condition, the object that was supposed to be passed was attached to the ground (the table). In the attached-to-ground control, the cord was anchored to the table, whereas the ball itself remained movable and could still be held in the beak. The purpose of this was to test whether subjects were able to discriminate between cases in which their body obstructed the lifting of the object and cases in which the lifting was obstructed for some other reason.

Each condition was tested in three sessions per individual, with 12 trials per session and a maximum time of 30 s per trial. For the pre-test only one session was done also with 12 trials per individual. The subjects participating in the experiment (N = 15) were divided into three groups of five each. Every group had the same number of trials and conditions, but the order of the conditions was counterbalanced. The subjects were not randomly assigned to the groups but selected based on prior task experience and expected performance. This was done to ensure that group allocation was as balanced as possible (group was included as a control factor in the analysis).

### Analysis

This experiment was filmed using two cameras (Nikon 1 V1 as the main camera and an iPhone camera as a backup), both positioned next to the table, where the experiment took place. The video material was then analyzed through the observation software BORIS with the help of an ethogram of the most prominent and relevant behaviors. All statistical analyses were conducted using R version 4.2.2 (R Core Team, 2022). We used binomial generalized linear mixed models (GLMMs) to test whether the probability of stepping off the mat differed across conditions. The response variable was Stepout (yes/no), with Condition (test, attached-to-ground, unattached) as the fixed test predictor, Group as a control predictor, and Subject as a random effect. We ran this model once on all sessions and once on Session 1 only to assess whether the condition effect was already present at the outset. To test whether performance changed across sessions, we compared models fitted to all sessions with and without Session as a fixed effect using a likelihood-ratio test. Significant condition effects were followed by post hoc pairwise comparisons of estimated marginal means. Because the primary question concerned whether birds stepped off the mat rather than when they did so, step-off occurrence within each 30-s trial was treated as the primary response, whereas total trial duration was analyzed separately as a secondary measure of performance efficiency.

## Supplementary Information

Supplementary Information 1.

Supplementary Video 1.

## Data Availability

The datasets generated and/or analyzed during the current study are available from the corresponding author on reasonable request.
