# Supplementary material for: Body awareness in Goffin’s cockatoos (Cacatua goffiniana)
Source: Sci Rep. 2026 Sep 17;16:28981. doi: 10.1038/s41598-026-57500-7 (PMC13582876; doi:10.1038/s41598-026-57500-7)
Supplement: Supplementary file 1 [file 41598_2026_57500_MOESM1_ESM.docx]

Body Self-Awareness in Goffin’s cockatoos (Cacatua goffiniana)

Paula Hundegger1, Remco Folkertsma1, Rachel Dale2, Alice M. I. Auersperg1, Antonio J. Osuna-Mascaró*,1

1Messerli Institute, University of Veterinary Medicine, 1210 Vienna, Austria.

2 Department for Psychosomatic Medicine and Psychotherapy, University for Continuing Education Krems, Austria.

Author for correspondence:

*Alice Auersperg

e-mail: alice.auersperg@vetmeduni.ac.at

Supporting Information

Supplementary Video Legend

Supplementary Video S1. Examples of the setup and procedure during session 1 in test condition, attached-to-ground control, and unattached control.
